# Explosive Disease Progression After Single-Agent B-cell Maturation Antigen-Targeted Treatment in Multiple Myeloma: A Report of Three Cases in Sheikh Shakhbout Medical City

**DOI:** 10.7759/cureus.44433

**Published:** 2023-08-31

**Authors:** Waed Jaber, Ammar Abdaljalil, Aya Ali, Mohamed Abu Haleeqa, Kayane Mheidly

**Affiliations:** 1 Department of Medicine, Division of Hematology, Sheikh Shakhbout Medical City, Abu Dhabi, ARE; 2 Department of Pharmacy, Sheikh Shakhbout Medical City, Abu Dhabi, ARE; 3 College of Medicine, The University of Jordan, Amman, JOR

**Keywords:** dreamm-2 trial, b-cell maturation antigen, extramedullary multiple myeloma, progression, relapsed & refractory multiple myeloma, belantamab mafodotin

## Abstract

Patients with “penta-refractory” multiple myeloma (MM) are challenging to treat given the limited treatment options available to them. Belantamab mafodotin is the first B-cell maturation antigen (BCMA)-targeting antibody-drug conjugate approved for the treatment of relapsed/refractory MM (RRMM). In this case report, we reviewed in detail three female patients who were diagnosed with MM international scoring system (ISS)-3 and were heavily pretreated, and refractory to CD38 monoclonal antibodies, two proteasome inhibitors, and two immunomodulatory agents. These patients were started on belantamab mafodotin and experienced rapid and explosive clinical, biochemical, and extramedullary disease progression within a short period of time. All three patients experienced worsening cytopenia, increased transfusion requirement, severe uncontrolled bony pain, recurrent infections, and frequent hospital admissions. Two of them passed away due to disease progression complications within a few months of starting belantamab mafodotin. Although belantamab mafodotin as a single agent was withdrawn from the market after the DREAMM-3 trial failed to achieve its primary endpoint in late RRMM, BCMA-targeted therapy may still be a promising treatment approach, and the role of belantamab mafodotin is yet to be revealed in combination therapy in early RRMM.

## Introduction

Multiple myeloma (MM) remains a heterogeneous incurable disease. Although its treatment paradigms and outcomes have progressed significantly, with roughly 18 newly approved treatments for MM in the past 12 years [1], it remains challenging to treat MM patients, as the disease can be resistant to standard-of-care treatments. Novel agents such as monoclonal antibodies have considerably improved outcomes in MM patients, and highly effective triplet regimens incorporating monoclonal antibodies, lenalidomide, and bortezomib are now used in early lines of treatment for some patients [2]. However, patients with “penta-refractory” disease, defined as a disease refractory to CD38 monoclonal antibodies (anti-CD38 mAb), two proteasome inhibitors (PIs), and two immunomodulatory agents (IMiDs), have poor outcomes with a median overall survival (OS) of 5.6 months [3]. Therefore, finding the optimal treatment for these patients represents a critical challenge, particularly given the more limited treatment options available to them.

Several emerging therapies are promising and show improved outcomes in patients with refractory and heavily pretreated MM. Belantamab mafodotin is the first B-cell maturation antigen (BCMA)-targeting antibody-drug conjugate approved for the treatment of relapsed/refractory MM (RRMM) [4]. BCMA, which is crucial for plasma cell proliferation and survival, is an antigen expressed on the malignant plasma cell surface at a much higher level compared to other cell types. Therefore, clinical trials are currently assessing the efficacy of using BCMA-targeted treatment strategies with chimeric antigen receptor T-cell lymphocyte products and antibody-drug conjugates. For example, the United States Food and Drug Administration (FDA) accelerated its approval for belantamab mafodotin based on the phase II DREAMM-2 trial, which assessed the safety and efficacy of two doses of the drug in RRMM patients [5].

The phase III DREAMM-3 trial was a randomized head-to-head superiority trial of belantamab mafodotin monotherapy versus pomalidomide in combination with low-dose dexamethasone in patients with relapsed or refractory MM. The trial did not meet its primary endpoint of progression-free survival (PFS) and overall survival (OS). Therefore, the FDA recommended the withdrawal of marketing authorization for belantamab mafodotin in patients with RRMM with at least four previous treatment lines [6]. Other trials under the DREAMM clinical trial program are currently set to assess the efficacy of belantamab mafodotin in combination regimens [7].

In this case report, we reviewed three patients with heavily treated RRMM who received belantamab mafodotin and ended up with explosive biochemical and extramedullary disease relapse.

## Case presentation

Patient 1

A 47-year-old female patient was diagnosed with MM international scoring system (ISS)-3 IgA kappa in 2008. Limited details were available about her initial diagnosis and treatment. She was treated initially with four cycles of thalidomide and dexamethasone followed by autologous stem cell transplant (ASCT) in June 2009 (Table 1).

**Table 1:** Patient demographics, disease characteristics, and treatment summary ISS: international scoring system; VRd: Velcade, Revlimid, and dexamethasone; VTD: bortezomib, thalidomide, dexamethasone; ASCT: autologous stem cell transplantation; DPd: daratumumab, pomalidomide, dexamethasone; DKd: daratumumab and dexamethasone;  PCd: pomalidomide-cyclophosphamide-dexamethasone; KRd: carfilzomib and lenalidomide plus dexamethasone; VCD: bortezomib, cyclophosphamide, and dexamethasone

Patient	Gender	Age at the time of starting belantamab (years)	Race	Date of diagnosis	Presentation	ISS	M Protein	Cytogenetics	Treatments
Patient 1	Female	60	South Asian	2008	Unknown	3	IgA Kappa	Not Available	Td, VRd, VTD, ASCT, DPd, DKd, PCd, Bendamustine & dexamethasone, Belantamab
Patient 2	Female	71	Southeast Asian	2018	Symptomatic anemia and bone pain	3	lambda light chain	1q21: Gain of CKS1B; 13q14: Deletion of RB1locus; 14q32: Loss of IGH	VRd, KRd, Maintenance Carfilozomib, DPd, DKd, belantamab
Patient 3	Female	65	Arab	2018	Back pain	3	lambda light chain	Absence of deletion p53, absence of IGH-FGFR3	VRd, VCD,Daratumumab, DPd, KRd, Kd, Belantamab

In 2013, her disease relapsed. She was then started on Velcade, Revlimid, and dexamethasone (VRd). After completing six cycles of VRd, disease evaluation showed stringent complete remission. A second ASCT was done in September 2013, followed by maintenance thalidomide until she relapsed again in 2017. She was started on daratumumab, pomalidomide, and dexamethasone (DPd), which was continued until October 2020.

In October 2020, the patient had an extramedullary disease relapse, as a neck-to-pelvis CT scan revealed plasmacytoma-like lesions in the right sacrum and right femur neck. Diffuse bony lytic lesions were also seen. A bone marrow biopsy on September 28, 2020, showed no increase in plasma cells. The sample sent for fluorescence in situ hybridization (FISH) had Insufficient plasma cells for further analysis. Carfilzomib was added to daratumumab and dexamethasone (DKd) as per the CANDOR trial [8], in addition to palliative radiation therapy to the right hip and iliac crest. A computed tomography (CT) scan indicated a good response from the bony lesions after four treatment cycles.

By October 2021, the patient completed eight cycles of DKd, and her myeloma biochemical markers started to increase (IgA kappa band concentrations increased from 1.2 g/L in June 2021 to 29.3 g/L in October 2021). The treatment was changed to pomalidomide, cyclophosphamide, and dexamethasone (PCd), but she developed a significant allergic reaction to pomalidomide four days after starting treatment, and it was stopped. Cyclophosphamide and dexamethasone were continued for three cycles; IgA kappa band further increased to 45.9 g/L, and the patient developed cytopenia and worsening transfusion requirement.

After the patient’s symptoms had worsened, selinexor and belantamab mafodotin were considered, but none of these agents were available in the UAE at that time. Instead, she was started on bendamustine and dexamethasone. She completed four cycles, with frequent blood and platelet transfusion support. Her M-protein level then remained stable.

In June 2022, after a multidisciplinary team discussion, they decided to treat this heavily treated RRMM with belantamab mafodotin, as it had become available in the UAE. She received two cycles of treatment, with a dose of 1.9 mg/kg in the first cycle, as the patient was frail, and 2.5 mg/kg in the second cycle. She continued to have a worsening transfusion requirement (three to four times per week) and recurrent hospital admissions, including to the intensive care unit to treat infections, a decreased level of consciousness, bleeding, hypercalcemia, and severe bone pain, which required daily follow-up from her pain-management team. She was also noticed to have skin lesions on her right arm with suspicion of myeloma deposits (Table 2).

**Table 2:** Disease assessment at diagnosis, before, and after starting belantamab mafodotin

	Patient 1			Patient 2			Patient 3		
	At time of Diagnosis	Before first dose of belantamab mafodotin	After last dose of blentamab mafodotin	At time of diagnosis	Before first dose of belantamab mafodotin	After last dose of blentamab mafodotin	At time of diagnosis	Before first dose of belantamab mafodotin	After last dose of blentamab mafodotin
Total Serum protein electrophoresis - (g/L)	Not available	112	108	57	57	Not available	103	62	92
Free light chain- kappa (mg/L)	Not available	14	16.4	2.27	8.75	Not available	6.44	Not available	2.49
Free light chain - lambda(mg/L)	Not available	2.03	4.67	1,770	657	Not available	3,100	Not available	499
Free kappa lambda ratio	Not available	6.9	3.51	0	0.01	Not available	0	Not available	0
IgG (g/L)	Not available	3.1	4.3	56.25	36.8	63.7	56.25	36.8	63.7
IgM (g/L)	Not available	0.07	0.18	0.09	0.06	0.06	0.09	0.06	0.06
IgA (g/L)	Not available	2.95	2.65	0.1	0.06	0.04	0.1	0.06	0.04
Bone marrow biopsy	Not available	Not available	Not available	Hypercellular marrow involved by lambda-restricted plasma calls.	Not available	Not available	Normocellular marrow, with massive replacement by monotypic lambda light chain restricted plasma cell infiltrates.	Not available	Not available
Bone Imaging	Not available	Multiple lytic lesion throughout the skull vault, destructive bone lesion involving medial aspect right ilium, old healed fracture/lesions RIGHT inferior and superior pubic rami	Interval development of bilateral enlarged cervical lymph node; mediastinal and upper abdominal, and retro peritoneal lymph nodes; soft tissues density seen along the presacral region; redemonstration of multiple osseous punched-out lytic lesions.	Soft tissue destructive lytic lesion involving the pedicle of T12 as well as upperend plates of the lumbar vertebra and pelvic/sacral bone.	Multiple lucent lytic lesions noted in bilateral sacrum, ileum, pubic bones, and visible proximal femur	Numerous widespread bone lesion with pathological fractures of left sixth rib	No lytic or blasting lesions seen on complete osseous survey	Multiple lytic lesion seen in ribs, spine, and scapula, large lytic lesion in the left scapula. Expansive lytic lesion seen in the right second and left 11th ribs, multiple healing fracture of the ribs bilateral.	Multiple lytic lesion throughout the thoracic and shoulder girdle. Appearance of numerous soft tissue masses a long the bilateral thoracic osseous wall and subcutaneous tissue with interval development of multiple bilateral lung nodules.

Due to the rapid deterioration in her performance status and worsening in her cytopenia with the related complications, belantamab mafodotin was stopped. A palliative treatment approach with supportive transfusion and optimization of pain management was then considered. The patient was transferred to long-term care facility, as she was not fit to be discharged, and passed away in September 2022.

Patient 2

A 66-year-old female patient was diagnosed to have MM ISS-3 lambda light chain in May 2018 (Table 1). At the same time, the patient was diagnosed with ampullary adenocarcinoma of the pancreas while being investigated for gastrointestinal blood loss as a cause of their anemia. She was treated with the Whipple procedure on June 6, 2018.

An initial diagnostic bone marrow biopsy on May 23, 2018, showed hypercellular bone marrow (100% cellularity) with markedly suppressed trilineage hematopoiesis and complete involvement of lambda light chain restricted plasma cells. A FISH study demonstrated a plasma cell population with a repeated CKS1B locus at 1q21, a loss of the RB1 locus at 13q14, and a loss of the IGH locus at 14q32. Such findings are often associated with high-risk diseases [9,10]. Further treatment was delayed until after the patient recovered from the Whipple procedure.

The patient was started on VRD on June 25, 2018. Dexamethasone was stopped during the first cycle and not received in the second cycle due to surgical site infection. After completing four cycles of VRD, a disease assessment did not show any improvement in the serum-free lambda light chain level.

In October 2018, the patient was started on second-line KRd, ultimately completing 12 cycles. Her free light chain ratio normalized, and serum protein electrophoresis (SPEP) detected no M-protein bands. The patient also had a worsening of their creatinine level and mild elevation in their liver enzymes, which improved after dose interruptions of carfilozomib and lenalidomide.

In January 2020, the patient was started on maintenance carfilozomib. By August 2020, the patient had completed five cycles of maintenance chemotherapy. Serum protein electrophoresis (SPEP) revealed an IgG lambda band detected in the gamma region with a protein level of 1.2 g/L. A bone marrow biopsy was performed on August 10, 2020, which showed bone marrow involvement with the patient’s known myeloma (60-70% of plasma cells according to CD138 immunohistochemistry and up to 80% according to a manual differential count). The patient complained of right shoulder pain, and X-rays revealed diffuse multiple lytic bone lesions consistent with their known history of MM.

Due to disease progression, DPd was initiated in November 2020. After three cycles, a bone marrow biopsy on January 31, 2021, revealed normocellular marrow (40-50% cellularity) with trilineage hematopoiesis and 7% lambda light chain restricted abnormal plasma cells. The sample sent for FISH had Insufficient plasma cells for further analysis. These results were consistent with a very good partial response, as per the International Myeloma Working Group [11]. The patient received a total of nine cycles of DPd treatment. After the eighth cycle, their serum-free lambda light chain level started to increase. Due to worsening bone pain, a whole-body CT scan was performed, which revealed numerous lytic lesions involving the spine, pelvic bones, and sternum.

Due to disease progression, the patient was started on DKd in August 2021, completing 14 cycles. She also received palliative radiotherapy to the right shoulder due to progressive lytic lesions and worsening pain. The serum-free lambda light chain level and lactate dehydrogenase were noticed to progressively increase. Due to worsening back pain, MRI of the whole spine was performed, which showed significant interval progression of the disease, with multiple focal enhancing lesions throughout the spine, multiple new lesions in the spinous processes of multiple vertebral bodies, and new lesions in the bilateral mandible and right paralaryngeal space.

The patient relapsed and their disease progressed after four different regimens of chemotherapy, including anti-CD38 mAb, two PIs, and two IMiDs. Therefore, belantamab mafodotin was discussed with the patient, and the first cycle was initiated in September 2022. Cardiac and ophthalmic assessments were done before starting belantamab mafodotin, and the patient was cleared to proceed. On physical examination, two scalp lesions were noticed and suspected to be myeloma deposits.

After completing two cycles of belantamab mafodotin, the patient’s bone pain worsened, their scalp lesions were noticed to increase in size, and their serum-free light chain level remained high but stable. A total of four cycles of belantamab mafodotin (2.5 mg/kg/dose) were completed by November 2022. The patient continued to require recurrent hospital admissions to control their severe bone pain. The patient suffered multiple pathological fractures; their performance status progressed to 2, and they became dependent on a wheelchair for mobilization. As imaging, including X-rays and CT scans, revealed disease progression, belantamab mafodotin was stopped (Table 2). In December 2022, the patient was switched to a palliative treatment approach, with dexamethasone (four days on and four days off), pain management, and supportive care.

Patient 3

A 61-year-old female patient was diagnosed with MM ISS-3 IGG lambda in May 2018 (Table 1). SPEP showed a distinct, dense, well-defined monoclonal band (Table 2). A bone marrow biopsy done in August 2018 indicated normocellular bone marrow for her age, displaying normal maturing trilineage hematopoiesis and massive replacement by monotypic lambda light chain restricted plasma cell infiltrates, consistent with plasma cell myeloma. A FISH study did not indicate TP53 deletion nor IGH-FGFR3 fusion.

She was started on VRd. However, she developed grade 3 allergic reaction to lenalidomide. Therefore, she was switched to bortezomib, cyclophosphamide, and dexamethasone (VCD), which started in December 2018 and lasted until May 2019. The VCD treatment was followed by bortezomib maintenance from May 2019 until September 2020, after which a disease assessment showed new lesions at the second and third lumbar vertebrae (L2-L3). As a result, DPd was started, which continued until January 2021. She was lost in follow-up and off treatment due to insurance and financial issues until September 2021, when new lytic lesions were seen on imaging.

Although the patient was scheduled to restart DPd, pomalidomide was initially not approved by her insurance company. Therefore, she received single-agent daratumumab starting in October 2021. She received a total of seven doses of daratumumab. She was then started on DPd, with a reduced dose of pomalidomide. After four cycles of DPd, her myeloma biomarkers were the same, with no significant improvement, and her treatment was switched to KRd, with a reduced dose of lenalidomide. During the second cycle of KRd, she developed a grade 3 allergic reaction to lenalidomide, which was treated with intravenous steroids, topical steroids, and an oral antihistamine. Lenalidomide was stopped. She received a total of five cycles of carfilzomib and dexamethasone, with a reduced dose of carfilzomib due to renal impairment.

At that time, the patient complained of worsening left shoulder pain. A spine and chest CT scan revealed multiple lytic lesions seen in the ribs, spine, and scapula (Table 2). The patient was treated with palliative radiation therapy to the left supra-clavicular fossa and cervical spine. Chemotherapy was interrupted due to coronavirus disease 2019 (COVID-19) infection, which was complicated by preserved ejection fraction heart failure.

The patient then was noticed to have new multiple skin lesions that were suspicious for plasmacytoma. As this result indicated disease progression despite using four chemotherapy regimens, including daratumumab-based and carfilzomib-based regimens, she was started on belantamab mafodotin in October 2022. She received a total of three doses (2.5 mg/kg/dose) with a regular follow-up with ophthalmology prior to each dose.

The patient course got complicated with recurrent hospital admissions secondary to infections, including viral acute bronchitis, urinary tract infection, hospital-acquired pneumonia, and sepsis. She continued to complain of fatigue, severe bone pain, and decreased oral intake, and her performance status progressed to 3. A chest X-ray revealed newly developed rib expansile lesions on either side in keeping with the progression of her disease. The skin lesions were also noticed to increase in size and number over a few weeks.

**Figure 1:**
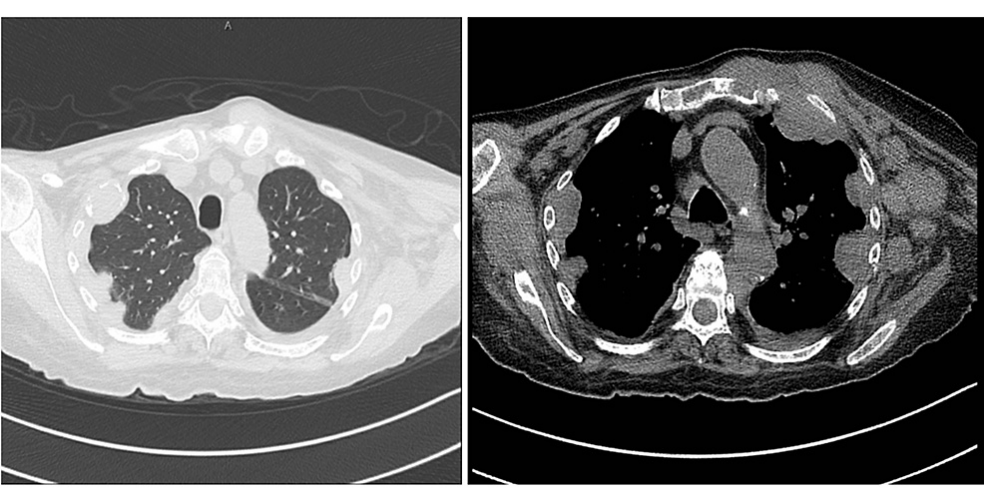
Chest CT scan showing numerous soft tissue masses along the bilateral thoracic osseous wall and subcutaneous tissues with interval development of multiple bilateral lung nodules, multiple lytic lesions throughout the thoracic wall (Patient 3)

In December 2022, the patient was admitted to the hospital with respiratory distress, increasing oxygen requirement, epistaxis, and spontaneous ecchymosis. A chest CT scan revealed significant disease progression compared to the previous CT scan done in August 2022 (Figure 1). Due to florid disease progression and rapid worsening of clinical status, belantamab was stopped, and a palliative treatment approach with oral dexamethasone and oral melphalan was started. The patient further deteriorated and passed away in December 2022.

## Discussion

In late RRMM and rapidly progressing disease, after three lines of therapy, including anti-CD38 mABs, the patient cannot wait for the manufacturing process of chimeric antigen receptor T-cell therapy (CAR-T) to be completed, which could take a few weeks; CAR-T cells are also not available in some countries. Belantamab mafodotin BCMA-targeted therapy has been considered a potential “off-the-shelf” drug that can be administered immediately in an outpatient setting. Real-world data have reported outcomes comparable with the DREAMM-2 trial in terms of objective response rate (33-45%), median progression-free survival (PFS) (2-6.5 months), and median overall survival (OS) (6-18 (not-reached) months) [12-14]. However, we did not see a similar response in our patients, as they did not respond to belantamab mafodotin treatment, and their disease rapidly progressed. The total number of cycles of belantamab mafodotin treatment ranged from two to four in our three reported cases .

A potential mechanism of belantamab mafodotin was recently reported by Matula et al., who assessed the effect of belantamab mafodotin on primary myeloma-stroma co-cultures [15]. It was noticed that the MM BCMA expression level positivity varies between 31% and 96% among patients, and inadequate expression is associated with extremely high resistance to belantamab mafodotin. Moreover, BCMA-negative myeloma subclones or myeloma subclones with low BCMA expression can develop alternative pathways to survive without BCMA. In addition, MMs become more resistant to belantamab mafodotin by enhancing the incorporation of mitochondria from autologous bone marrow stromal cells (BM-MSCs), which is similar to previously reported resistance mechanisms of other medications, such as the PI, carfilzomib, or the BCL-2 inhibitor, venetoclax. BM-MSCs somehow support the survival and delay the apoptosis of malignant plasma cells in the presence of belantamab mafodotin.

The persistence of the BCMA antigen on MM cells is crucial for the efficacy of belantamab mafodotin. Zhou et al. suggested that monitoring tumor BCMA levels might help to assess BCMA expression status in deciding the next appropriate therapeutic approach for MM patients, as it is possible for the BCMA antigen to be downregulated or partially lost with targeted therapies [16]. As belantamab mafodotin use as a single agent was withdrawn from the market after the DREAMM-3 trial failed to achieve its primary endpoint in late RRMM, the role of belantamab mafodotin in combination therapy in early RRMM has yet to be revealed [17-19].

Ocular adverse drug reaction necessitates frequent and close ophthalmology monitoring and may lead hemato-oncologists and patients to consider options other than belantamab when choosing a regimen in early RRMM. The use of belantamab mafodotin may be supported once additional mitigation strategies of ocular toxicity are proved to be worthy of use, including alternate dosing schedules and improved supportive care, as the mechanistic details of belantamab mafodotin-associated keratopathy become better understood.

Although our case series had a small number of patients, it still represented a similar population sample compared with patients included in the DREAMM-2 trial [5]. This patient population had a heavily pretreated and aggressive RRMM; they also exhausted multiple lines of treatment and remained challenging to manage. Thus, they represented our real-life experience, as well as our struggles in treating such patients.

This case series was limited in that patients were treated in other facilities or even other countries at some point in their disease course, with incomplete information about their initial diagnostic workup, especially cytogenetics (Case 1), treatment decisions, and disease assessment (with unknown clonal evolution) during these periods of time. Furthermore, there were insurance and financial issues that resulted in treatment interruptions and incomplete work-up for some signs and symptoms of the disease. Patients’ lack of access to new treatment modalities such as CAR T-cell therapy, bispecific antibody therapy, and cereblon E3 ligase modulators, among many other options, as well as a lack of access to clinical trials, may also limit options to address unmet needs in such patient populations.

## Conclusions

We noticed rapid disease progression in heavily pretreated MM patients after starting belantamab mafodotin; despite this, BCMA-targeted therapy may still be a promising treatment approach. Further studies are needed to determine the best way to integrate belantamab mafodotin into the standard of care for RRMM. Introducing belantamab mafodotin at an earlier stage or combining it with agents that target other antigens are some of the suggested strategies to overcome possible belantamab mafodotin resistance due to probable loss of the target antigen.

Single-agent belantamab mafodotin was granted accelerated approval by the FDA as a monotherapy to treat patients with RRMM who have received at least four prior therapies, based on the results of the phase II DREAMM-2 trial. However, the phase III randomized DREAMM-3 trial failed to meet its primary endpoints of PFS and OS. Medications that receive accelerated approval based on phase II trials should be closely reviewed and used with caution. Treatment decisions should rely on phase III trials, which provide more reliable information about medication efficacy.
